# Antiviral screening of natural, anti-inflammatory compound library against African swine fever virus

**DOI:** 10.1186/s12985-024-02374-2

**Published:** 2024-04-25

**Authors:** Joshua A. Jackman, Astghik Hakobyan, Rafayela Grigoryan, Roza Izmailyan, Charles C. Elrod, Hovakim Zakaryan

**Affiliations:** 1 School of Chemical Engineering and Translational Nanobioscience Research Center, Sungkyunkwan University, Suwon, 16419 Republic of Korea; 2 Laboratory of Antiviral Drug Discovery, Institute of Molecular Biology of NAS, Yerevan, Armenia; 3 Natural Biologics Inc, Newfield, NY 14867 USA; 4 Department of Animal Science, Cornell University, Ithaca, NY 14853 USA

**Keywords:** African swine fever virus, Antiviral, Anti-inflammatory, Cytokine production, Screening

## Abstract

**Background:**

African swine fever virus (ASFV) is a major threat to pig production and the lack of effective vaccines underscores the need to develop robust antiviral countermeasures. Pathologically, a significant elevation in pro-inflammatory cytokine production is associated with ASFV infection in pigs and there is high interest in identifying dual-acting natural compounds that exhibit antiviral and anti-inflammatory activities.

**Methods:**

Using the laboratory-adapted ASFV BA71V strain, we screened a library of 297 natural, anti-inflammatory compounds to identify promising candidates that protected Vero cells against virus-induced cytopathic effect (CPE). Virus yield reduction, virucidal, and cell cytotoxicity experiments were performed on positive hits and two lead compounds were further characterized in dose-dependent assays along with time-of-addition, time-of-removal, virus entry, and viral protein synthesis assays. The antiviral effects of the two lead compounds on mitigating virulent ASFV infection in porcine macrophages (PAMs) were also tested using similar methods, and the ability to inhibit pro-inflammatory cytokine production during virulent ASFV infection was assessed by enzyme-linked immunosorbent assay (ELISA).

**Results:**

The screen identified five compounds that inhibited ASFV-induced CPE by greater than 50% and virus yield reduction experiments showed that two of these compounds, tetrandrine and berbamine, exhibited particularly high levels of anti-ASFV activity. Mechanistic analysis confirmed that both compounds potently inhibited early stages of ASFV infection and that the compounds also inhibited infection of PAMs by the virulent ASFV Arm/07 isolate. Importantly, during ASFV infection in PAM cells, both compounds markedly reduced the production of pro-inflammatory cytokines involved in disease pathogenesis while tetrandrine had a greater and more sustained anti-inflammatory effect than berbamine.

**Conclusions:**

Together, these findings support that dual-acting natural compounds with antiviral and anti-inflammatory properties hold promise as preventative and therapeutic agents to combat ASFV infection by simultaneously inhibiting viral replication and reducing virus-induced cytokine production.

**Supplementary Information:**

The online version contains supplementary material available at 10.1186/s12985-024-02374-2.

## Background

African swine fever virus (ASFV) is the etiological agent of African swine fever (ASF), which is a highly infectious hemorrhagic disease of domestic pigs and wild boars with mortality rates close to 100% [1, 2]. ASF causes heavy losses to the pork industry of affected countries due to the lack of effective vaccines [3, 4]. In 2018, ASF outbreaks were reported in China and neighboring countries that are collectively responsible for almost half of the world’s pork production, thereby presenting a global threat to the pork supply [5]. This issue has led to strict biosecurity practices to prevent ASF spread as well as the search for natural compounds with antiviral properties that can mitigate ASFV in potential transmission vectors such as water and feed [6–8]. In addition to these preventative measures, there is high interest in discovering natural compounds that might also be capable of curbing ASFV infection in pigs based on inhibiting viral genome replication along with reducing disease symptoms such as inflammation [9].

Pigs infected with highly virulent ASFV strains display high fever, with temperatures up to 42 °C, lethargy, inactivity, and anorexia [10]. The affected animals usually show cyanosis, nasal discharges, vomiting, and diarrhea. Animals die within 7 days from the onset of disease. Hemorrhagic splenomegaly and lymphadenitis are recorded during *post-mortem* observations. Hemorrhagic lesions may also be observed with less frequency in other organs. While various pathogenic mechanisms have been proposed as primary contributors to hemorrhages, the current consensus identifies pro-inflammatory cytokine production as the initial cause of lesions in ASF [11, 12]. Serum analysis of infected pigs revealed a significant elevation of pro-inflammatory cytokines, including TNF-α, IFN-α, IL-1β, IL-6, IL-8, and IL-12, which is commonly referred to as “cytokine storm” [13]. The “cytokine storm” effect is common for several highly pathogenic viruses such as SARS-CoV-2, influenza virus, and Ebola virus, and can result in multiple organ failure and mortality [14]. Thus, controlling pro-inflammatory cytokine production may prevent disease progression and reduce the mortality rate associated with ASF and other viral diseases.

Previously, the antiviral efficacy of some natural compounds possessing generally recognized anti-inflammatory properties was studied against ASFV. For instance, we have reported that the flavonoid apigenin significantly inhibited ASFV replication in immortalized Vero cells [15]. Genkwanin, a natural derivative of apigenin, also exerted potent anti-ASFV activity in Vero cells and porcine macrophages (PAMs) [16]. These results prompted us to consider the potential of other anti-inflammatory natural compounds as inhibitors of ASFV infection. Such compounds may have the potential to act as dual-purpose antiviral drugs, which are capable of not only inhibiting ASFV replication but also mitigating the “cytokine storm” associated with ASFV infection.

Herein, we applied a small library screening approach to evaluate the antiviral activity of 297 natural compounds with generally recognized anti-inflammatory properties. The screen was conducted using the laboratory-adapted, non-virulent ASFV BA71V strain [17] and led us to identify five natural compounds that inhibited virus-induced cytopathic effect (CPE) by greater than 50%. Virus yield reduction experiments identified that two of these anti-inflammatory compounds, tetrandrine and berbamine, exhibited particularly high levels of anti-ASFV activity. The antiviral effects of tetrandrine and berbamine on mitigating virulent ASFV infection in PAMs were also confirmed. Notably, during ASFV infection in PAM cells, both compounds, especially tetrandrine, markedly reduced the production of pro-inflammatory cytokines such as TNF-α that plays a key role in ASF disease pathogenesis [18].

## Methods

### Cells, viruses, and compounds

Eagle’s minimum essential medium (EMEM; Lonza, Switzerland) with 10% fetal bovine serum (Lonza, Switzerland), 2 mM L-glutamine (Lonza, Switzerland), 100 IU mL^− 1^ penicillin (Reyoung, China), and 100 µg mL^− 1^ streptomycin (Arterium, Ukraine) were used to grow Vero (African green monkey kidney) cells at 37 °C. The antiviral screening process and some antiviral assays were conducted by using the Vero-adapted ASFV BA71V strain as specified. We measured the titer of the Vero-adapted ASFV BA71V strain by cytopathic effect (CPE) assay on Vero cells. We used the Spearman-Kärber endpoint method to calculate the titer, which was expressed in log TCID_50_ mL^− 1^ units.

Experiments with the highly virulent ASFV Arm/07 isolate involved porcine alveolar macrophages (PAMs) that were prepared as previously reported [19]. The porcine macrophages were maintained at 37 °C in Dulbecco’s modified Eagle’s medium (Sigma-Aldrich, Germany) that was supplemented with 10% fetal bovine serum, 2 mM L-glutamine, 100 IU mL^− 1^ penicillin, and 100 µg mL^− 1^ streptomycin. Titration of the ASFV Arm/07 isolate was conducted using the hemadsorption (HAD) assay and expressed in log HADU_50_ mL^− 1^ units.

A library comprising 297 natural compounds (Additional file 1. Table S1) was obtained from ChemFaces (China). The compounds were selected based on their known anti-inflammatory properties and undocumented efficacy against ASFV. Each compound was dissolved in dimethyl sulfoxide (DMSO) as a 5 mg mL^− 1^ stock solution and diluted in EMEM. Dilutions in the cell culture medium were made to maintain a final DMSO concentration below 1% (v/v).

### Library screening

Confluent Vero cells were seeded in 96-well cell culture plates at a density of 2 × 10^4^ cells per well. The cells were subsequently infected with the ASFV BA71V strain (at 0.2 TCID_50_ per cell) and immediately treated with test compounds at a concentration of 50 µM, except for some compounds that were tested at 25 µM due to cytotoxic effects (see Table S1) for 72 h at 37 °C. Following the incubation period, once complete CPE (as indicated by cell rounding, detachment, and extensive destruction of cell monolayer) was observed in the untreated wells (Additional file 1. Fig. S1), cell viability was assessed using the MTT assay. In brief, Vero cells were washed with cold PBS and MTT solution [3-(4,5-dimethylthiazol-2-yl)-2,5-diphenyltetrazolium bromide from Sigma-Aldrich, Germany] was added. The cells were then allowed to incubate at 37 °C for 2 h, followed by the extraction of purple formazan using the MTT test solvent (DMSO). The optical density (OD) was measured colorimetrically at 570 nm by using a microplate reader (BioTek Epoch 2, Agilent Technologies, USA). The percentage of CPE inhibition was calculated by the following formula: (OD_*tv*_-OD_*v*_/OD_*c*_-OD_*v*_) × 100% where OD refers to the optical density of each well, *tv* refers to ASFV-infected cells treated with compound, *c* refers to mock-infected cells without treatment, and *v* refers to ASFV-infected cells without treatment.

ASFV-infected cells treated with a minimal concentration of DMSO (< 0.5%) were utilized as the negative control, while ASFV-infected cells treated with 50 µM of apigenin were used as the positive control. Each plate contained six samples of both negative and positive controls. The OD values obtained from these controls were used to calculate the Z’-factor, which is a statistical parameter to assess the variability of the screening assay [20]. The Z’-factor was calculated by using the following formula: 1-[(3σ_*p*_ + 3σ_*n*_)/(µ_*p*_-µ_*n*_)], where σ refers to the standard deviation, µ refers to the mean, *p* refers to the positive control, and *n* refers to the negative control. If the Z’-factor value is between 0.5 and 1.0, then the screening assay performance is considered consistent and robust.

### Compound validation assays

For the yield reduction assay, Vero cells were cultivated in 24-well cell culture plates with a seeding density of 2 × 10^5^ cells per well. The cells were subsequently exposed to the ASFV BA71V strain (at 0.2 TCID_50_ per cell) and treated with selected compounds at concentrations of 50 µM or 25 µM (for tetrandrine). Following a 72-h incubation period, the supernatant was harvested and titrated.

For the virucidal assay, a virus suspension containing 2 × 10^5^ TCID_50_ ASFV particles was incubated with selected compounds at indicated concentrations for 1 h at room temperature. Then, Vero cells seeded in 96-well cell culture plates (seeding density: 2 × 10^4^ cells per well) were exposed to 20-fold dilutions of the treated viral suspension to eliminate potential virostatic effects of the test compounds on ASFV infection. After a 24-h post-infection, the supernatant was harvested and subjected to titration.

### Dose-dependent assay

Vero cells (2 × 10^5^ cells per well) or PAMs (4 × 10^4^ cells per well) cultured in 24-well plates were subjected to infection with the ASFV BA71V strain [multiplicity of infection (MOI) of 0.2 TCID_50_] or ASFV Arm/07 isolate (MOI of 0.5 HADU_50_), respectively. Following infection, cells were treated with selected compounds at different concentrations in a two-fold dilution format. The infection was allowed to progress for 24 h, after which the supernatant was harvested and titrated.

### MOI-dependent assay

Vero cells cultured in 24-well plates (2 × 10^5^ cells per well) were infected with the ASFV BA71V strain at different MOI values ranging from 0.1 to 1 TCID_50_ per cell and treated with selected compounds at indicated concentrations. The virus was collected and titrated at 24 h post-infection.

### Virus replication stage and treatment assays

For the ASFV replication cycle experiments, Vero cells (2 × 10^5^ cells per well) cultured in 24-well plates were infected with the ASFV BA71V strain (MOI of 0.2 TCID_50_) and then treated with selected compounds at indicated concentrations. The infection was allowed to progress for 24, 48, or 72 h, after which the supernatants were harvested and titrated.

For time-of-addition experiments, Vero cells (2 × 10^5^ cells per well) or PAM cells (4 × 10^4^ cells per well) were grown in 24-well cell culture plates. The wells were marked as − 2, 0, 2, 8, and 16 h, corresponding to the time period relative to the onset of ASFV infection (inoculation). These time points cover the early, middle, and late stages of ASFV infection during one replication cycle. In the pre-infection format, Vero cells or macrophages were exposed to the selected compounds before (− 2 h) infection with the ASFV BA71V strain (0.2 TCID_50_ per cell) or ASFV Arm/07 isolate (0.5 HADU_50_ per cell). In the co-infection format, Vero cells were simultaneously exposed to compounds and ASFV. In the post-infection format, cells were infected with ASFV and the compounds were then added at 2, 8, or 16 h post-infection. The supernatants were collected at 24 h post-infection and titrated.

For time-of-removal experiments, Vero cells grown in 24-well cell culture plates were infected with the ASFV BA71V strain and treated with compounds at indicated concentrations. Afterwards, the compound was removed after incubation at 2-, 4-, 8-, 12-, or 16-h post-treatment. This removal step was done by washing the cells with 1× PBS and replacing the media. The supernatant was then collected and titrated 24 h post-infection.

### Virus entry assays

In the virus attachment experiments, Vero cells (2 × 10^5^ cells per well) or macrophages (4 × 10^4^ cells per well) were grown in 24-well cell culture plates. The cells were incubated with the ASFV BA71V strain (0.2 TCID_50_) or ASFV Arm/07 isolate (0.5 HADU_50_), respectively, along with the test compounds at 4°C for 1 h. This step was done to facilitate virus binding while preventing internalization. Following this procedure, the unbound virus and compound were removed by thoroughly washing the cells with 1× PBS. Fresh medium containing 3% FBS was then added. Virus in the supernatant was collected and titrated 24 h post-infection.

In the internalization experiments, Vero cells or macrophages were infected with ASFV BA71V or ASFV Arm/07, respectively, at 4 °C for 1 h. Following this step, unbound virus was removed by thoroughly washing the cells with 1× PBS. The compounds were then added to the cells and incubated at 37 °C, allowing virus entry to proceed for 1 h. Subsequently, the compounds were removed by washing with 1× PBS to prevent their effect on later stages of infection. The infection was then allowed to continue for 24 h, after which the supernatants were collected and titrated.

### Cytotoxicity assay

The cytotoxicity of natural compounds was examined in Vero cells and PAMs by using the crystal violet staining method [21]. Cells (2 × 10^4^ cells per well) reached confluence and were then exposed to increasing compound concentrations, which ranged from 3.1 µM to 50 µM. The cells were incubated for 72 h at 37 °C in 5% CO_2_. Following incubation, the cell culture medium was discarded and a 4% crystal violet solution (in ethanol) was added to the wells. The solution was allowed to incubate for 40 min at room temperature. Then, the wells were washed with distilled water, and 200 µL of methanol was added to each well for 20 min. The OD of each well was measured at 570 nm using a plate reader (BioTek Epoch 2, Agilent Technologies, USA). The relative viability of the treated cells compared to mock-treated cells (no compound) was expressed as a percentage and calculated at each concentration using the following formula: OD_*t*_/OD_*c*_ × 100%, where OD_*t*_ and OD_*c*_ correspond to the absorbance of treated and negative control (mock-treated) cells, respectively.

### Western blotting

Vero cells (5 × 10^5^ cells per well) were grown in 6-well cell culture plates and infected with ASFV at a MOI of 2 TCID_50_ in the presence or absence of test compounds at two different compound concentrations. After 24 h, the cells were dissociated using Laemmli buffer and heated for 5 min at 95 °C, electrophoresed in sodium dodecyl sulfate-polyacrylamide gels, and transferred onto nitrocellulose membranes (GE Healthcare). The membranes were incubated with mouse monoclonal antibodies p30, p72, or tubulin (Sigma Aldrich) at dilutions of 1:1000, 1:1000, and 1:2000, respectively. Antibodies were detected using horseradish peroxidase (HRP) conjugated secondary antibodies. Bands were visualized by using a Chemidoc XRS imaging system from BioRad.

### Cytokine quantification

The release of TNF-α, IL-1β, and IL-6 cytokines into the culture medium was quantified using commercially available ELISA kits (R&D Systems, USA). Experiments were performed according to the instructions provided by the manufacturer. For each cytokine, a standard curve was plotted, which represented the cytokine concentration as a function of the OD value. The standard curve was used to derive a linear regression equation, which was then utilized to determine the concentration of each cytokine released.

### Statistical analysis

All experiments were conducted in triplicate. Data are expressed as mean ± standard deviation (s.d.) of three independent experiments. The unpaired Student’s t-test (versus virus-only positive control) and one-way analysis of variance (ANOVA) with Tukey’s multiple comparisons test (versus other test groups) were used for antiviral and anti-inflammatory experiments, respectively. Statistical significance was computed in terms of standard or multiplicity-adjusted *P* values as appropriate, and *P* < 0.05, *P* < 0.01, and *P* < 0.001 indicate the levels of statistical significance (*, **, ***) while ns indicates a non-significant effect (*P* > 0.05). The GraphPad Prism software program (version 10.0.2, GraphPad Software, USA) was used to calculate 50% inhibitory concentration (IC_50_) values by variable slope analysis (four-parameter logistic curve) of absolute virus quantities with appropriate units as a function of compound concentration. The IC_50_ values correspond to the compound concentrations needed to reduce viral infectivity by 50%.

## Results

### Antiviral screening of natural compound library

**Fig. 1:**
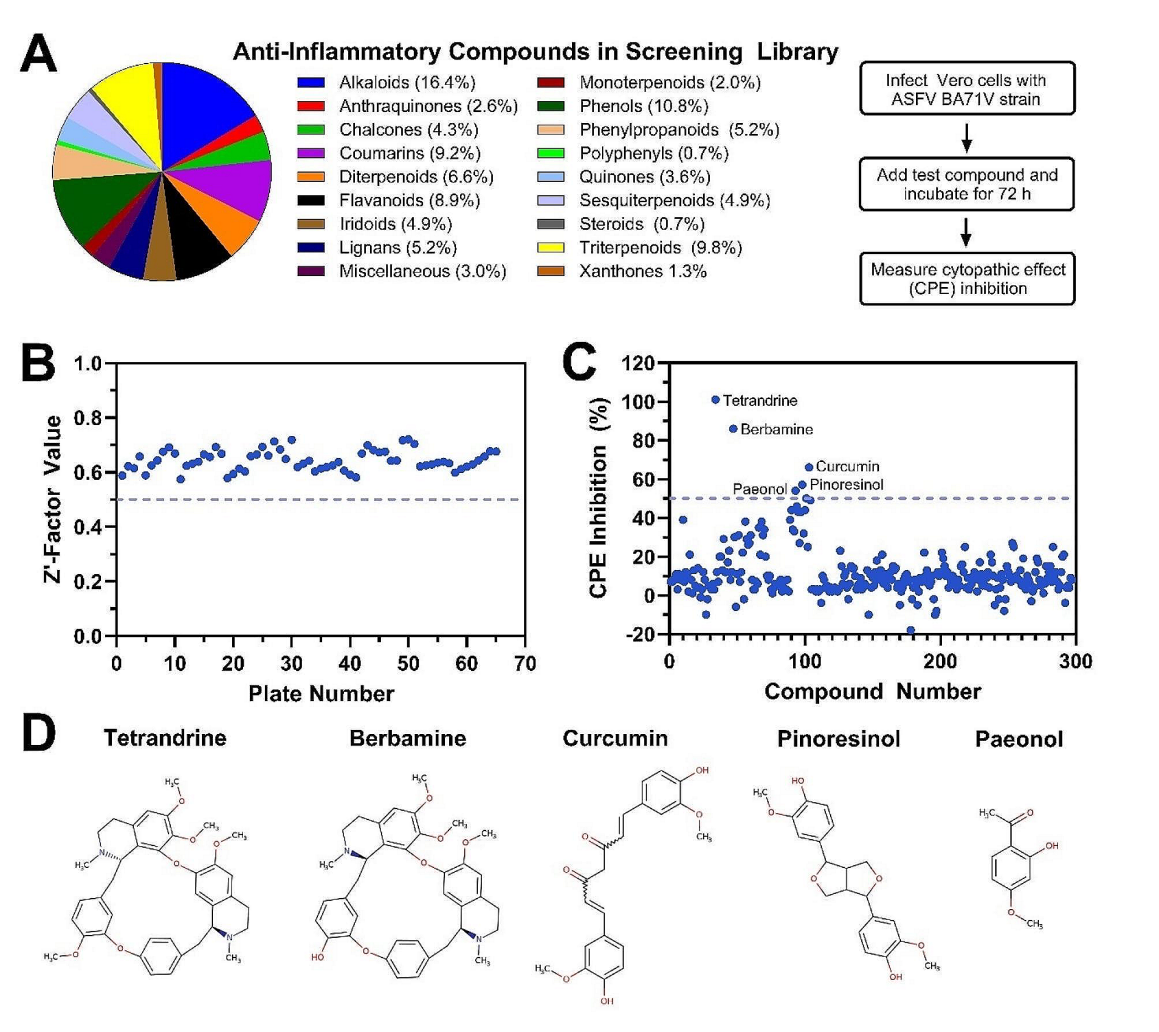
Screening of anti-inflammatory compound library for anti-ASFV activity. ASFV-infected Vero cells were incubated with each compound at the defined concentration and the inhibitory effect on virus-induced CPE was measured by MTT assay at 72 h post-infection. **(A)** Percentage of anti-inflammatory compounds in 297-member library belonging to different molecular classes and experimental steps. **(B)** Z’ score of each plate in the screen, which was calculated by using the minimum (negative control: DMSO) and maximum (positive control: apigenin) MTT signals. **(C)** Percentage inhibition of virus-induced CPE by tested compounds. Compounds that inhibited ASFV-induced CPE by ≥ 50% and did not damage cell monolayer were defined as positive hits. All experiments were conducted in triplicate and mean values are presented for clarity. **(D)** Five top-performing compounds selected for further mechanistic studies

We screened a 297-member library of natural compounds with anti-inflammatory properties (see Table S1). The compounds belong to 17 distinct classes of natural compounds (Fig. 1A). Alkaloids, phenols, and triterpenoids represented approximately 16%, 11%, and 10% of all tested compounds, respectively. The screening process was conducted by using a cell-based colorimetric assay in combination with the MTT method to track CPE development [22]. Compounds were tested at 50 µM concentrations, except for some compounds that were tested at 25 µM concentration due to cytotoxicity at higher concentrations (cf. Table S1). Each screening plate contained negative and positive control wells, which allowed us to calculate the maximum (positive control) and minimum (negative control) MTT optimal density values to generate Z’-factor values.

All 66 plates (*n* = 3) had Z’ scores higher than 0.5 and the lowest and highest Z’ score values of individual plates were 0.575 and 0.721, respectively (Fig. 1B). The average Z’ score value was 0.646, which indicated that the entire screening process had high consistency and was robust for the screening of anti-ASFV compounds [20]. To select hit candidates, we applied the following two criteria: (a) > 50% inhibition of ASFV-induced CPE; and (b) no destruction of cell monolayer upon treatment. Based on these criteria, five compounds, namely paeonol, pinoresinol, curcumin, berbamine, and tetrandrine, were selected based on CPE inhibition levels of 54%, 57%, 66%, 86%, and 101%, respectively (Fig. 1C). Paeonol and curcumin are both phenolic compounds, whereas pinoresinol is classified as a tetrahydrofuran lignan (Fig. 1D). By contrast, berbamine and tetrandrine have significantly more complex chemical structures than the other three compounds and are both classified as bis-benzylisoquinoline alkaloids.

### Validation of positive-hit compounds against ASFV

**Fig. 2:**
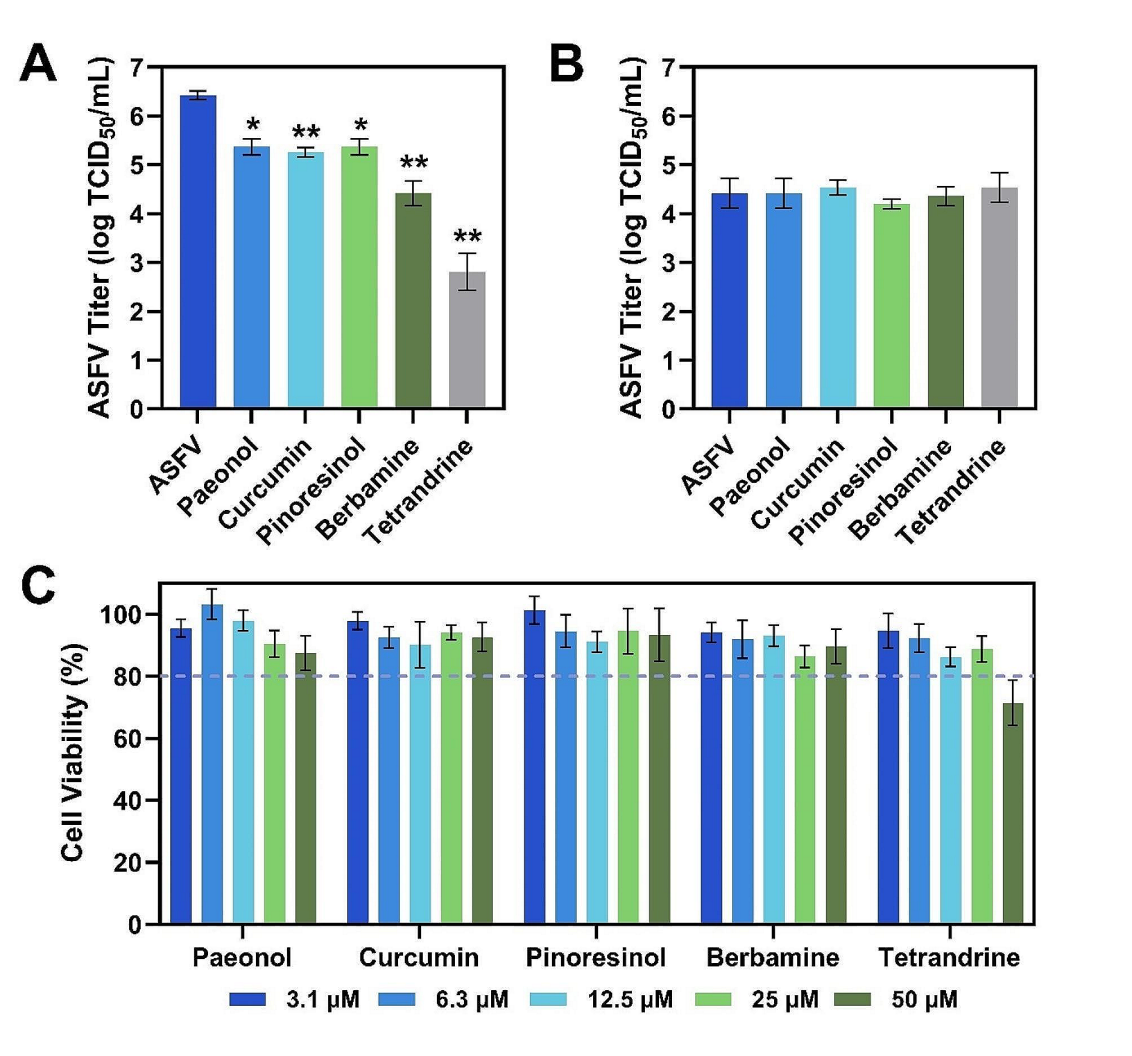
Antiviral validation of selected anti-inflammatory compounds. **(A)** Antiviral activity of compounds to inhibit ASFV infection in Vero cells. Virus and compound were added at the same time to cells and the viral titer in supernatant was measured. **(B)** Direct (virucidal) effect of compounds on extracellular ASFV particles. Virus and compound were pre-mixed, incubated, diluted 20-fold, and then the diluted virus-compound mixture was added to infect cells. The viral titer in supernatant was measured. **(C)** Vero cell viability after incubation with different compound concentrations, as evaluated by crystal violet staining method. The horizontal dashed line corresponds to a 20% drop in relative cell viability. The relative viability of mock-treated cells was defined as 100%. In panels (A) and (B), all compounds were tested at 50 µM except for tetrandrine, which was tested at 25 µM. ASFV means virus-only control without compound addition. Results represent the mean ± s.d. of three independent experiments (*n* = 3). Significant differences compared to control are denoted by ^*^p < 0.05 and ^**^p < 0.01

We conducted virus yield reduction experiments to further validate the antiviral effect of the selected five compounds. Vero cells were infected with ASFV and simultaneously exposed to compounds at 50 µM concentration, except for tetrandrine that was used at 25 µM concentration. The infection was allowed to progress for 72 h until complete CPE was observed in the negative controls (virus only), at which point the supernatant was harvested and titrated. All five compounds displayed statistically significant (*p* < 0.05) antiviral effects on ASFV replication in Vero cells (Fig. 2A). Tetrandrine significantly decreased the viral titer from 6.4 ± 0.1 log TCID_50_ mL^− 1^ to 2.8 ± 0.1 log TCID_50_ mL^− 1^, resulting in a 3.6 log reduction, and berbamine had the second greatest inhibitory effect. By contrast, paenol and pinoresinol exhibited the weakest inhibitory effects, reducing the viral yield by only 1 log, and curcumin had only a slightly greater inhibitory effect.

To further investigate whether the compounds exhibit a direct antiviral effect on ASFV, we also conducted a virucidal assay. The virus suspension was pre-incubated with compounds for 1 h and then the mixture was diluted 20-fold to sub-inhibitory concentrations before adding the diluted virus-compound mixtures to the Vero cells. At 24 h post-infection, the supernatant was collected and titrated. The test compounds did not exhibit virucidal activity (Fig. 2B). Therefore, the observed antiviral effects of these compounds cannot be attributed to a direct impact on ASFV particles. Furthermore, the compounds did not demonstrate cytotoxicity at tested concentrations, indicating that their antiviral effects were independent of cell toxicity (Fig. 2C). The two most potent compounds, berbamine and tetrandrine, were selected for further studies.

### Antiviral mechanism of action of berbamine and tetrandrine

**Fig. 3:**
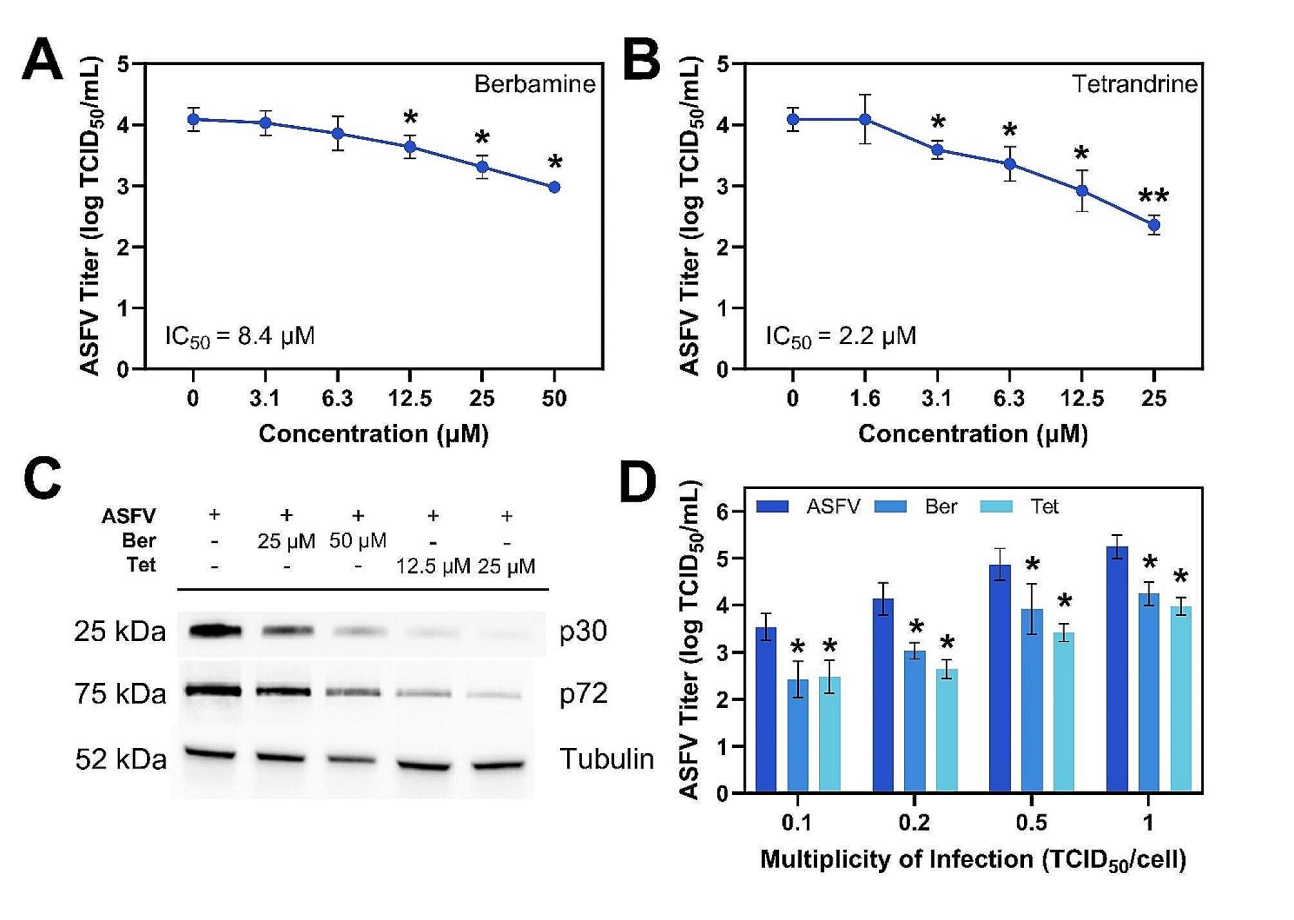
Antiviral activity of berbamine and tetrandrine against ASFV in Vero cells. **(A)** ASFV titer for cells exposed to berbamine at increasing concentrations from 3.1 µM to 50 µM. **(B)** ASFV titer for cells exposed to tetrandrine at increasing concentrations from 1.6 µM to 25 µM. **(C)** Western blot analysis of ASFV protein synthesis in ASFV-infected cells exposed to berbamine and tetrandrine at selected concentrations. Tubulin served as a loading control and cropping was done to show regions of interest. **(D)** Effect of berbamine and tetrandrine on Vero cells infected with ASFV at different MOIs. The concentrations of berbamine and tetrandrine for the experiments in panel (C) were 50 µM and 25 µM, respectively. ASFV means virus-only control without compound addition while Ber and Tet denote berbamine and tetrandrine, respectively. Results represent the mean ± s.d. of three independent experiments (*n* = 3). Significant differences compared to control are denoted by ^*^p < 0.05 and ^**^p < 0.01

Our initial evaluation of mechanistic aspects was carried out during a single ASFV replication cycle and virus was collected at 24 h post-infection, which corresponds to the time duration required for one full cycle of ASFV BA71V strain infection kinetics in Vero cells [23, 24]. First, we studied whether berbamine and tetrandrine may exhibit antiviral activity at lower compound concentrations. Vero cells infected with ASFV were treated with berbamine and tetrandrine at varying concentrations. The inhibitory effect of both compounds occurred in a dose-dependent manner (Fig. 3A, B). Statistically significant antiviral activity (*p* < 0.05) was observed at the lowest concentrations of 12.5 µM for berbamine and 3.1 µM for tetrandrine. The IC_50_ value for berbamine was calculated to be 8.4 µM, while the IC_50_ value of tetrandrine was 2.2 µM.

Dose-dependent antiviral effects were also observed when evaluating the synthesis of early (p30) and late (p72) ASFV proteins (Fig. 3C). Berbamine and tetrandrine significantly hampered the synthesis of p30 and p72 proteins at 50 µM and 25 µM concentrations, respectively, whereas the inhibitory effect was less pronounced at lower concentrations. Then, we studied the inhibitory effects of berbamine and tetrandrine on ASFV replication at different MOIs. Our findings revealed a significant reduction in viral yield following treatment with berbamine and tetrandrine at different MOIs, resulting in a decrease of approximately 1-1.1 log and 1.1–1.5 log, respectively (Fig. 3D). This result supports that the antiviral efficacy of these compounds is consistent regardless of the initial viral load infecting the cells.

**Fig. 4:**
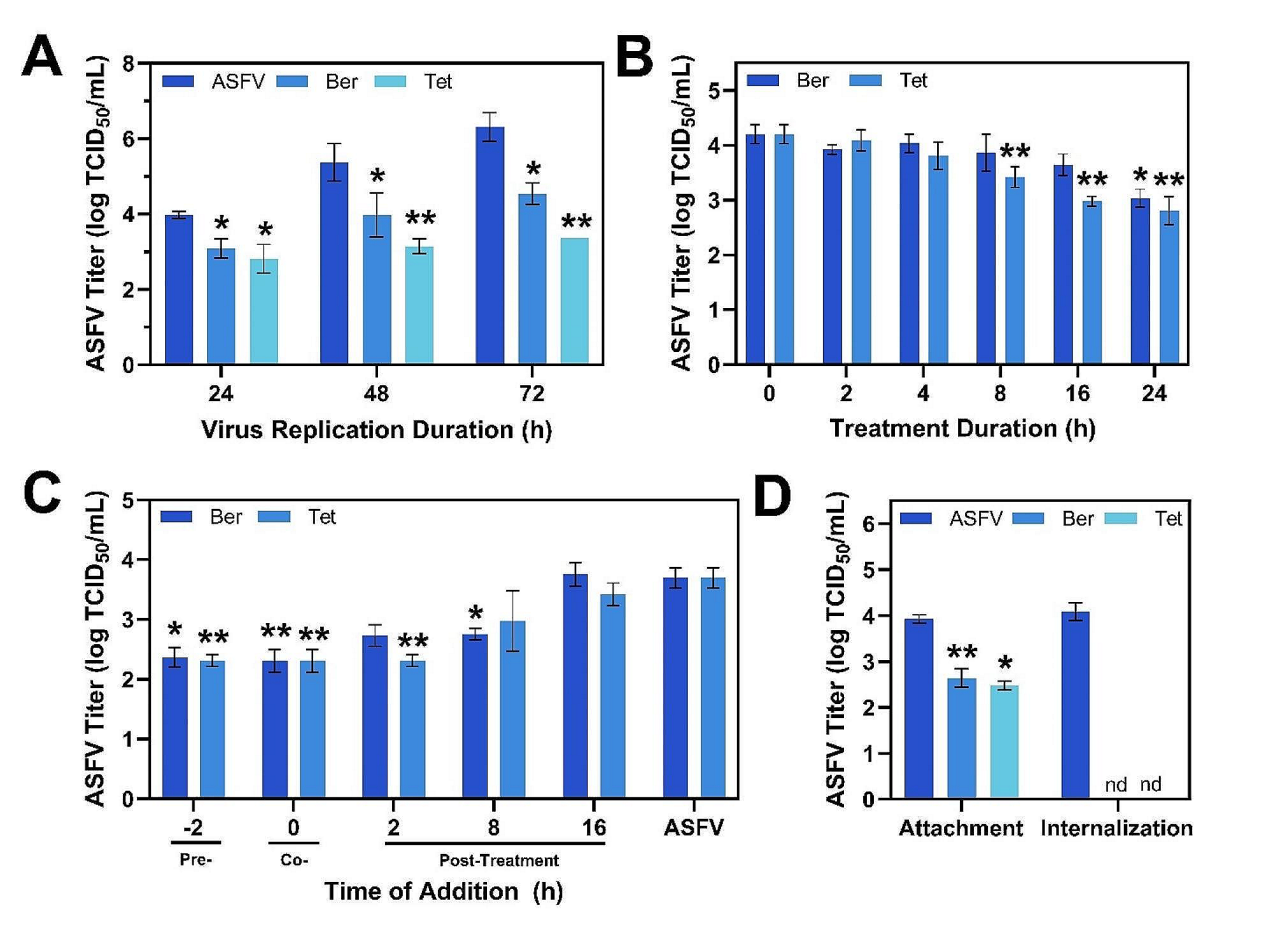
Mechanistic analysis of antiviral activity by berbamine and tetrandrine. **(A)** Antiviral activity of berbamine and tetrandrine to inhibit ASFV in Vero cells depending on the virus replication duration, as indicated by supernatant collection time post-infection. **(B)** Effect of compound removal time on antiviral activity in Vero cells. Initially, virus and compound were added at the same time to cells and removal was done by media exchange. **(C)** Antiviral activity depending on the time of addition. **(D)** Effect of berbamine and tetrandrine on ASFV attachment to and internalization by Vero cells. The concentrations of berbamine and tetrandrine in all experiments were 50 µM and 25 µM, respectively. ASFV means virus-only control without compound addition while Ber and Tet denote berbamine and tetrandrine, respectively. nd refers to not determined (below detection limit). Results represent the mean ± s.d. of three independent experiments (*n* = 3). Significant differences compared to control are denoted by ^*^p < 0.05 and ^**^p < 0.01

In our primary screen, we observed an ∼ 3 log reduction after treatment with 25 µM tetrandrine. However, during the single replication cycle experiments, we observed only a 1-log reduction. This difference led us to propose that tetrandrine can block ASFV replication in subsequent cycles. Indeed, the suppression of ASFV replication by berbamine and tetrandrine tended to be greater when the virus was collected at 72 h post-infection compared to 24- and 48-h post-infection (Fig. 4A). This finding indicates that both compounds exerted their antiviral effects across multiple rounds of virus replication.

As the greatest antiviral effect was observed at 72 h post-infection, we also studied the incubation duration required for 1-log (90% inhibition) reduction. For this purpose, berbamine and tetrandrine were added to ASFV-infected Vero cells at 0 h post-infection and removed by medium replacement after specific intervals. The virus was collected at 24 h post-infection and quantified. As expected, our results showed a gradual decrease in viral titers as the interval between infection and compound removal increased (Fig. 4B). Berbamine demonstrated a 1-log reduction in viral titer when ASFV-infected cells were exposed to this compound for 24 h. In contrast, tetrandrine achieved a similar level of viral titer reduction after 16 h of treatment.

Next, we determined the specific replication stages of ASFV infection that are affected by berbamine and tetrandrine. Compounds were added at five time points before and after infection, followed by virus collection and titration at 24 h post-infection. The most significant antiviral effects of berbamine and tetrandrine were observed when cells were pre-treated with compound or co-treated with compound alongside ASFV infection (Fig. 4C).

Berbamine reduced the viral yield from 3.98 ± 0.19 log TCID_50_ mL^− 1^ to 2.25 ± 0.09 log TCID_50_ mL^− 1^ (*p* < 0.01) when added at 0 h, in parallel to ASFV infection. Similarly, tetrandrine decreased the ASFV titer from 3.98 ± 0.19 log TCID_50_ mL^− 1^ to 2.31 ± 0.19 log TCID_50_ mL^− 1^ (*p* < 0.01) during co-addition. By contrast, there was no statistically significant antiviral effect on ASFV replication when berbamine and tetrandrine were added at 16 h post-infection. These results suggest that both compounds hamper ASFV replication by interfering with early stages of infection. Therefore, we further studied the effect of these compounds on the viral entry stage, which consists of virus attachment and internalization substages.

To investigate the effect of berbamine and tetrandrine on ASFV attachment to Vero cells, compounds were added at 4 °C, in which condition virus particles bind to but do not enter cells. Treatment with berbamine and tetrandrine inhibited virus particle attachment to Vero cells, resulting in a decrease of the ASFV yield from 3.92 ± 0.09 log TCID_50_ mL^− 1^ to 2.64 ± 0.09 log TCID_50_ mL^− 1^ (*p* < 0.01) and 2.47 ± 0.19 log TCID_50_ mL^− 1^ (*p* < 0.05), respectively (Fig. 4D). A more significant decrease was observed when compounds were added at the ASFV internalization stage. Berbamine and tetrandrine were added immediately after a temperature shift from 4 °C to 37 °C and removed after 1 h of treatment in order to minimize further antiviral effects on later stages of ASFV replication. Both compounds reduced the viral yield below the detection limit corresponding to < 2.2 log TCID_50_ mL^− 1^. Altogether, these results indicate that berbamine and tetrandrine inhibit ASFV infection by blocking virus entry.

### Antiviral activity of berbamine and tetrandrine in ASFV-infected PAMs

Monocytes and macrophages are the main target cells for ASFV replication in pigs. Since ASFV has different entry mechanisms into Vero cells and PAMs [25], we studied the antiviral activity of berbamine and tetrandrine in ASFV-infected PAMs as well. For these experiments, we used the highly virulent ASFV Arm/07 isolate that is capable of infecting PAMs and causes disease in pigs [26, 27]. First, we incubated ASFV-infected PAMs with increasing concentrations of compounds that were not toxic to PAMs within the testing range (Additional file 1. Fig. S2).

**Fig. 5:**
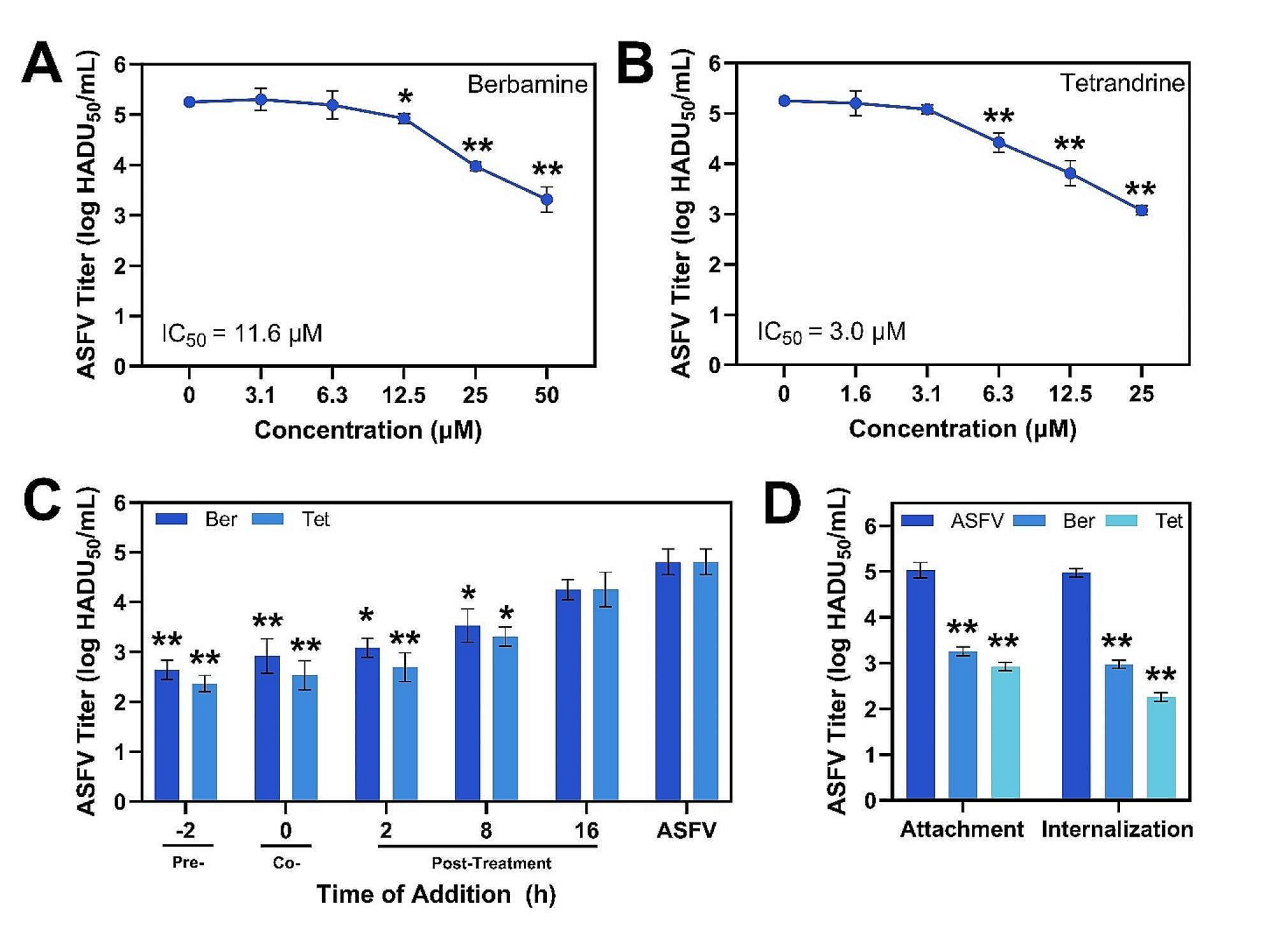
Antiviral activity of berbamine and tetrandrine against ASFV Arm/07 isolate in PAM cells. ASFV titer in cells exposed to different concentrations of **(A)** berbamine (3.1–50 µM) or **(B)** tetrandrine (1.6–25 µM). **(C)** Antiviral activity depending on the time of addition. **(D)** Effect of berbamine and tetrandrine on ASFV attachment to and internalization by PAM cells. The concentrations of berbamine and tetrandrine for the experiments in panels (C) and (D) were 50 µM and 25 µM, respectively. ASFV means virus-only control without compound addition while Ber and Tet denote berbamine and tetrandrine, respectively. Results represent the mean ± s.d. of three independent experiments (*n* = 3). Significant differences compared to control are denoted by ^*^p < 0.05 and ^**^p < 0.01

Both compounds decreased the viral titer in a dose-dependent manner and the corresponding IC_50_ values of berbamine and tetrandrine were 11.6 µM and 3.0 µM, respectively (Fig. 5A, B). Based on these initial results, we used the highest non-toxic concentrations (50 µM and 25 µM for berbamine and tetrandrine, respectively) in further time-of-addition and virus entry experiments. Time-of-addition experiments were conducted with the same five different time points before and after infection as in the case of the Vero cell experiments, and the results showed a similar pattern of inhibition (Fig. 5C). The most potent inhibitory effects were observed due to pre- and co-treatment of PAM cells with the compounds.

Then, we conducted experiments to reveal the effect of berbamine and tetrandrine on ASFV attachment to and internalization by PAMs. When berbamine and tetrandrine were added at the virus attachment substage, they decreased the ASFV yield from 5.03 ± 0.16 log HADU_50_ mL^− 1^ to 3.25 ± 0.09 log HADU_50_ mL^− 1^ (*p* < 0.01) and 2.92 ± 0.09 log HADU_50_ mL^− 1^ (*p* < 0.01), respectively (Fig. 5D). A more potent inhibitory effect was observed when the compounds were added at the internalization substage. Berbamine reduced the viral titer from 4.97 ± 0.09 log HADU_50_ mL^− 1^ to 2.97 ± 0.09 log HADU_50_ mL^− 1^, while tetrandrine also blocked ASFV internalization, resulting in a virus titer decrease from 4.97 ± 0.09 log HADU_50_ mL^− 1^ to 2.25 ± 0.09 log HADU_50_ mL^− 1^. Based on these results, we conclude that berbamine and tetrandrine exerted significant inhibition of ASFV infection by disrupting the viral entry process. This inhibition was observed independently from the host cell type or ASFV strain used in our experiments.

### Anti-inflammatory activity of berbamine and tetrandrine in ASFV-infected PAMs

As the natural compounds in the library were initially selected due to their anti-inflammatory properties, we also investigated the potential of berbamine and tetrandrine to reduce the expression of pro-inflammatory cytokines by ASFV-infected PAMs. While both compounds have generally recognized anti-inflammatory properties such as reducing lipopolysaccharide (LPS) stimulation of macrophages (see, e.g., Refs [28, 29]), their effects on curbing ASFV infection-related inflammation are unknown. As different immune-activating biological processes can involve various signaling pathways [30], it is thus important to evaluate anti-inflammatory effects in disease-specific contexts in terms of the etiological agent, cell type, and measured inflammation markers. Therefore, we quantified TNF-α, IL-1β, and IL-6 cytokine levels in PAM culture supernatants that were collected at 24- and 48-h post-infection using commercially available ELISA kits.

Following a similar protocol, our experimental approach was first validated by measuring cytokine levels in uninfected PAMs incubated with LPS (1 µg mL^− 1^) with or without berbamine or tetrandrine (Additional file 1. Fig. S3). The results demonstrated that both berbamine and tetrandrine significantly decreased TNF-α and IL-1β production in LPS-treated PAMs at both time points as expected. Of note, tetrandrine also exhibited significant inhibition of IL-6 production, whereas the effect of berbamine on that cytokine was not statistically significant.

We proceeded to test the anti-inflammatory properties of berbamine and tetrandrine in an ASFV-focused context by infecting PAMs with ASFV with or without berbamine or tetrandrine treatment before proceeding to measure cytokine production levels. In this format, the cytokine levels produced by PAMs increased following infection with the ASFV Arm/07 isolate, which is consistent with previously reported findings [31, 32]. In particular, the TNF-α, IL-1β, and IL-6 levels increased by around 18-, 11-, and 13-fold, respectively, at 24 h post-infection and by around 23-, 22-, and 21-fold, respectively, at 48 h post-infection compared to uninfected PAMs.

**Fig. 6:**
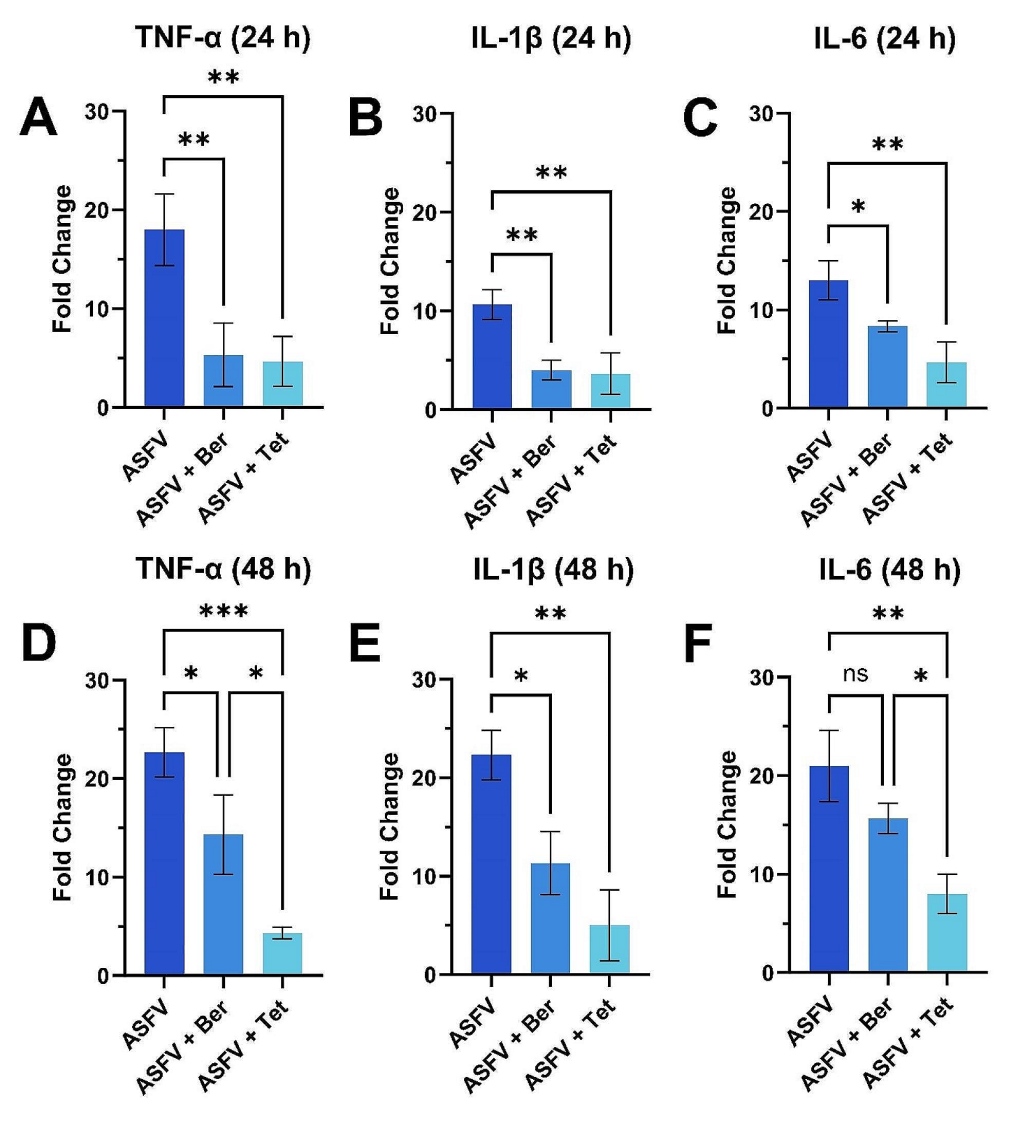
Inhibition of pro-inflammatory cytokine production in ASFV-infected PAMs. Effect of berbamine (Ber) or tetrandrine (Tet) on inhibition of **(A)** TNF-α, **(B)** IL-1β, and **(C)** IL-6 production in ASFV-infected cells at 24 h post-infection. **(D-F)** Corresponding measurements at 48 h post-infection. Cytokine measurements were performed by ELISA on collected supernatants. The concentration of berbamine and tetrandrine for the experiments were 50 µM and 25 µM, respectively. ASFV means virus-only control without compound addition while Ber and Tet denote berbamine and tetrandrine, respectively. Results represent the mean ± s.d. of three independent experiments (*n* = 3). Significant differences compared to other test groups are denoted by ^*^p < 0.05, ^**^p < 0.01, and ^***^p < 0.001

Importantly, berbamine and tetrandrine treatment had strong effects on inhibiting pro-inflammatory cytokine production compared to the levels found in untreated ASFV-infected cells. At 24 h post-infection, both berbamine and tetrandrine significantly reduced TNF-α, IL-1β, and IL-6 levels compared to the ASFV control by around 60–80% (Fig. 6A-C). Notably, at 48 h post-infection, both berbamine and tetrandrine significantly reduced TNF-α levels by around 37% and 81% respectively, while the inhibitory effect of tetrandrine was greater (*p* < 0.05) (Fig. 6D). Both compounds also reduced IL-1β levels at 48 h post-infection by around 49% and 78%, respectively, and the results were statistically similar in this case (Fig. 6E). Importantly, only tetrandrine significantly reduced IL-6 levels at 48 h post-infection by around 62%, whereas the effect of berberine was insignificant (Fig. 6F).

Thus, both compounds displayed anti-inflammatory activity in ASFV-infected PAMs while tetrandrine demonstrated a superior activity profile in terms of greater inhibitory levels and more sustained effect throughout the course of infection. Collectively, our findings demonstrate that both compounds, especially tetrandrine with its high effectiveness, possess the ability to not only impede ASFV replication but also to mitigate ASFV-induced production of clinically important pro-inflammatory cytokines.

## Discussion

ASFV remains a significant threat to the global swine industry and is characterized by its devastating impact on pig populations and the absence of approved vaccines or specific antiviral therapies [33]. The absence of preventive measures against ASFV highlights the critical need to discover effective interventions to control and manage this highly contagious disease in pigs. Within this context, the exploration of natural compounds with potential antiviral properties has gained immense importance and highlights the opportunity to identify novel inhibitory strategies to combat ASFV infection [9].

In this work, the screening of a library of natural compounds with anti-inflammatory properties led to the identification of five promising candidates—paeonol, pinoresinol, curcumin, berbamine, and tetrandrine—that showed substantial inhibition of ASFV-induced CPE without affecting cell monolayers. These compounds belong to distinct classes, with berbamine and tetrandrine exhibiting more complex chemical structures compared to the other lead candidates. Subsequent validation experiments in Vero cells confirmed the antiviral efficacy of these compounds against ASFV replication. Tetrandrine displayed the most potent effect, achieving a 3.6 log reduction in viral titer, while paeonol and pinoresinol exhibited weaker inhibitory effects. Notably, none of these five compounds demonstrated virucidal activity or cytotoxic effects at tested concentrations, confirming the specificity of their antiviral activities. Recently, Zhu et al. and Qian et al. independently reported the antiviral activity of berbamine and tetrandrine against ASFV in vitro [34, 35], thereby additionally validating the robustness of our screening results.

In the present context, further mechanistic investigations focused on berbamine and tetrandrine revealed their dose-dependent antiviral activities, inhibiting the synthesis of ASFV proteins and consistently reducing viral yields across different MOIs. Time-of-addition experiments elucidated the compounds’ action points within the viral replication cycle, highlighting their significant impact during pre- and co-treatment phases. Berbamine and tetrandrine significantly inhibited ASFV replication by interfering with early infection stages, particularly virus attachment and internalization into host cells, that in turn disrupted viral entry. Interestingly, both compounds exhibited sustained antiviral effects over multiple replication cycles (cf. Figure 4A). This finding fits with the relatively long half-lives that have been reported for both compounds [36], which supports their capability to impact ASFV replication over several cycles. Given that both compounds also exhibited antiviral activity during early stages of viral infection post-internalization in the present experiments (cf. Figure 4C), it is reasonable that the most significant antiviral effect was observed during the prolonged presence of compounds in the cell culture medium (cf. Figure 4B) and the results are consistent with their effects on virus replication at various stages, especially viral entry.

Furthermore, extending the study to primary target cells, porcine macrophages, revealed consistent antiviral effects of berbamine and tetrandrine. Notably, these compounds inhibited ASFV infection by disrupting viral entry mechanisms, suggesting that their antiviral mechanism is irrespective of host cell type or virus strain. It has been reported that tetrandrine inhibits Ebola virus entry into human monocyte-derived macrophages by disrupting endosomal Ca^2+^ channels [37]. In addition, berbamine suppresses the replication of Japanese encephalitis virus by blocking Ca^2+^ permeable non-selective cation channels in endosomes [38]. Therefore, it is reasonable to hypothesize that comparable inhibitory mechanisms might play a role in exerting antiviral effects against ASFV. Conducting further experiments could potentially unveil the involvement of endosomal Ca^2+^ channels in ASFV infection in future work.

Importantly, we also demonstrated that berbamine and tetrandrine effectively reduced the production of pro-inflammatory cytokines, namely TNF-α, IL-1β, and IL-6, in ASFV-infected macrophages, which had not been explored before. Among these cytokines, TNF-α is considered to play an essential role in driving pathological changes observed in ASFV-infected pigs [13, 39]. Therefore, compounds such as berbamine and tetrandrine can not only suppress viral replication but also demonstrate the capability to mitigate pathological conditions associated with the heightened production of pro-inflammatory cytokines due to viral infection. Of note, tetrandrine exhibited a particularly high and sustained level of anti-inflammatory activity in the ASFV infection context and, together with its high antiviral activity, supports that it has promising merits.

These findings are particularly significant because berbamine and tetrandrine, along with other bis-benzylisoquinoline alkaloids, have also been recently reported to inhibit porcine epidemic diarrhea virus (PEDV) with similar potency [40, 41], suggesting their broad utility as porcine virus countermeasures. At the same time, previous studies have focused on validating the antiviral activity of these compounds and did not evaluate potential infection-related anti-inflammatory effects. Our findings expand on this viewpoint and demonstrate proof-of-principle to show that the anti-inflammatory properties of berbamine and tetrandrine are translatable to inhibiting production of pro-inflammatory cytokines during viral infection along with reducing viral load.

## Conclusions

In conclusion, our results indicate that berbamine and tetrandrine possess dual inhibitory activities: potent antiviral activity against ASFV, primarily through disrupting viral entry mechanisms; and significant inhibition of virus-induced pro-inflammatory cytokine production. Thus, these compounds hold promise as potential preventative and therapeutic agents to combat ASFV infection, with tetrandrine exhibiting particularly robust antiviral and anti-inflammatory properties that warrant further investigation and development.

### Electronic supplementary material

Below is the link to the electronic supplementary material.

Additional file 1: Fig. S1. Representative image of complete CPE developed in Vero cells after infection with ASFV at 72 h post-infection. Fig. S2. Effect of berbamine and tetrandrine on PAM cell viability. Fig. S3. Inhibition of pro-inflammatory cytokine production in LPS-challenged PAMs. Table S1. Natural, anti-inflammatory compounds that were tested against ASFV

## Data Availability

The datasets used and/or analyzed during the current study are available from the corresponding authors on reasonable request.

## Abbreviations

ASFV: African swine fever virus
Berbamine: Ber
CPE: Cytopathic effect
EMEM: Eagle’s minimum essential medium
ELISA: Enzyme-linked immunosorbent assay
HADU_50_: 50% hemadsorption dose units
LPS: Lipopolysaccharide
MOI: Multiplicity of infection
MTT: 3-(4,5-dimethylthiazol-2-yl)-2,5-diphenyltetrazolium bromide
OD: Optical density
PAM: Porcine macrophage
PBS: Phosphate-buffered saline
PEDV: Porcine epidemic diarrhea virus
TCID_50_: 50% tissue culture infective dose
Tetrandrine: Ter
