# Supplementary material for: Antiviral screening of natural, anti-inflammatory compound library against African swine fever virus
Source: Virol J. 2024 Apr 25;21:95. doi: 10.1186/s12985-024-02374-2 (PMC11046949; doi:10.1186/s12985-024-02374-2)
Supplement: Supplementary file 1 — Additional file 1: Fig. S1. Representative image of complete CPE developed in Vero cells after infection with ASFV at 72 h post-infection. Fig. S2. Effect of berbamine and tetrandrine on PAM cell viability. Fig. S3. Inhibition of pro-inflammatory cytokine production in LPS-challenged PAMs. Table S1. Natural, anti-inflammatory compounds that were tested against ASFV [file 12985_2024_2374_MOESM1_ESM.docx]

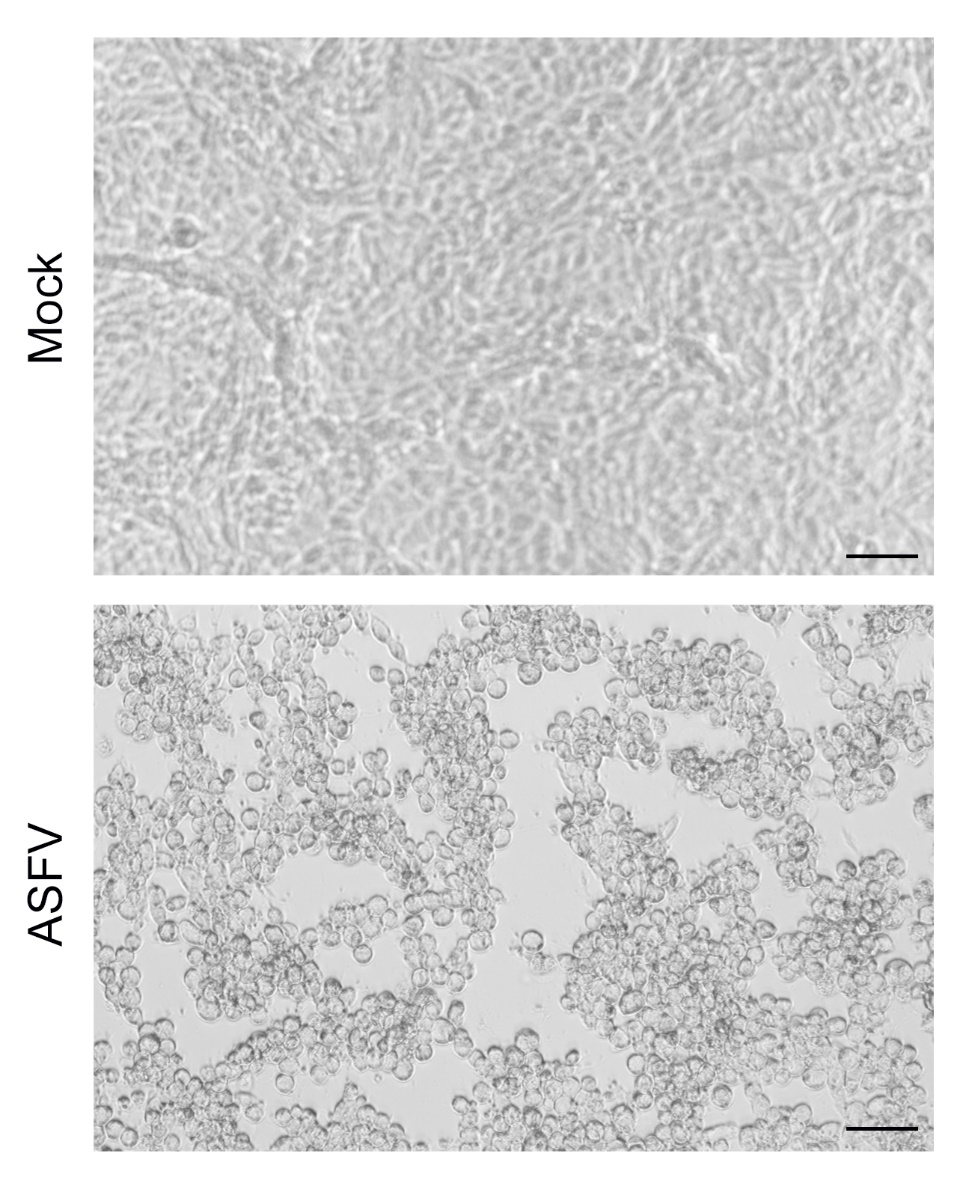


**Fig. S1.** Representative image of complete CPE developed in Vero cells after infection with ASFV at 72 h post-infection. An image of mock-infected Vero cells is presented for comparison. Scale bars are 100 µM.


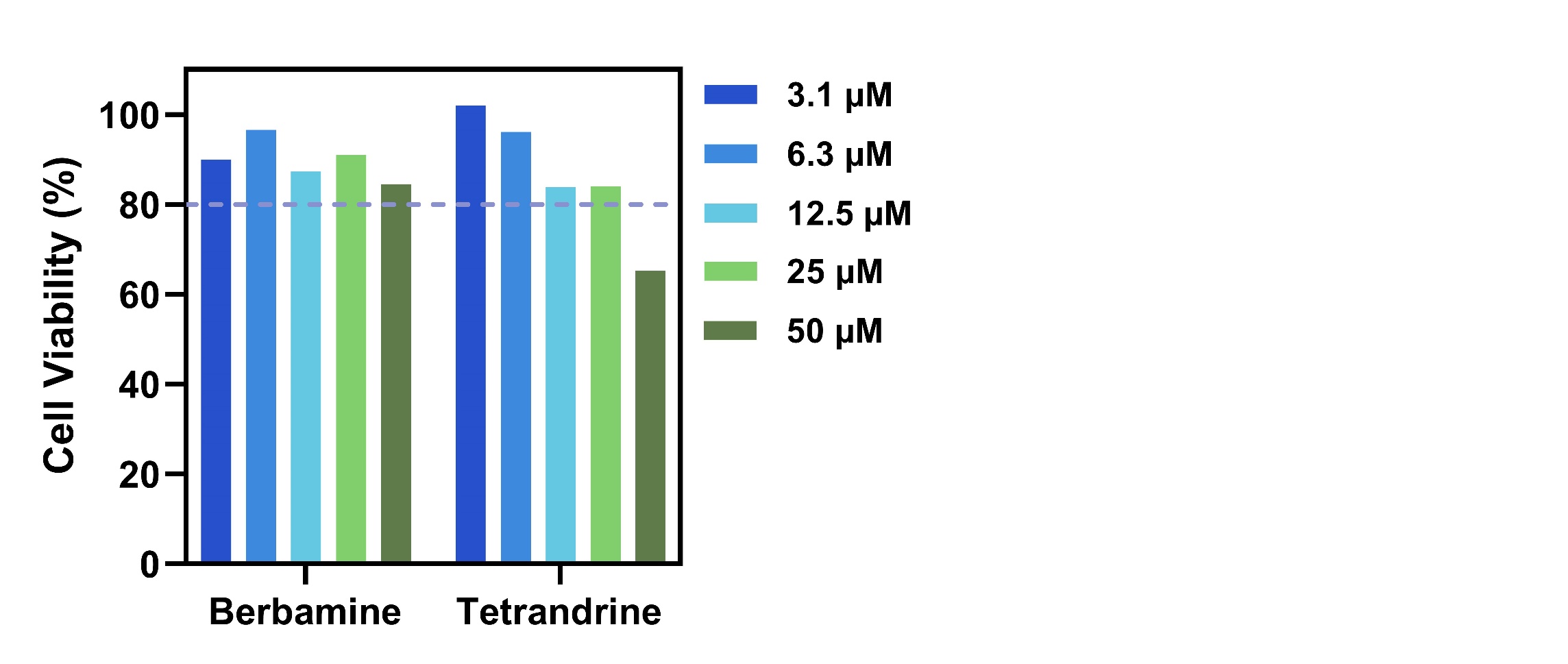


**Fig. S2.** Effect of berbamine and tetrandrine on PAM cell viability. After cell incubation with different compound concentrations, the crystal violet method was used to measure relative cell viability compared to that of mock-treated PAM cells. The horizontal dashed line corresponds to a 20% drop in relative cell viability. The relative viability of mock-treated cells was defined as 100%. Results represent the mean of two independent experiments (*n*=2).


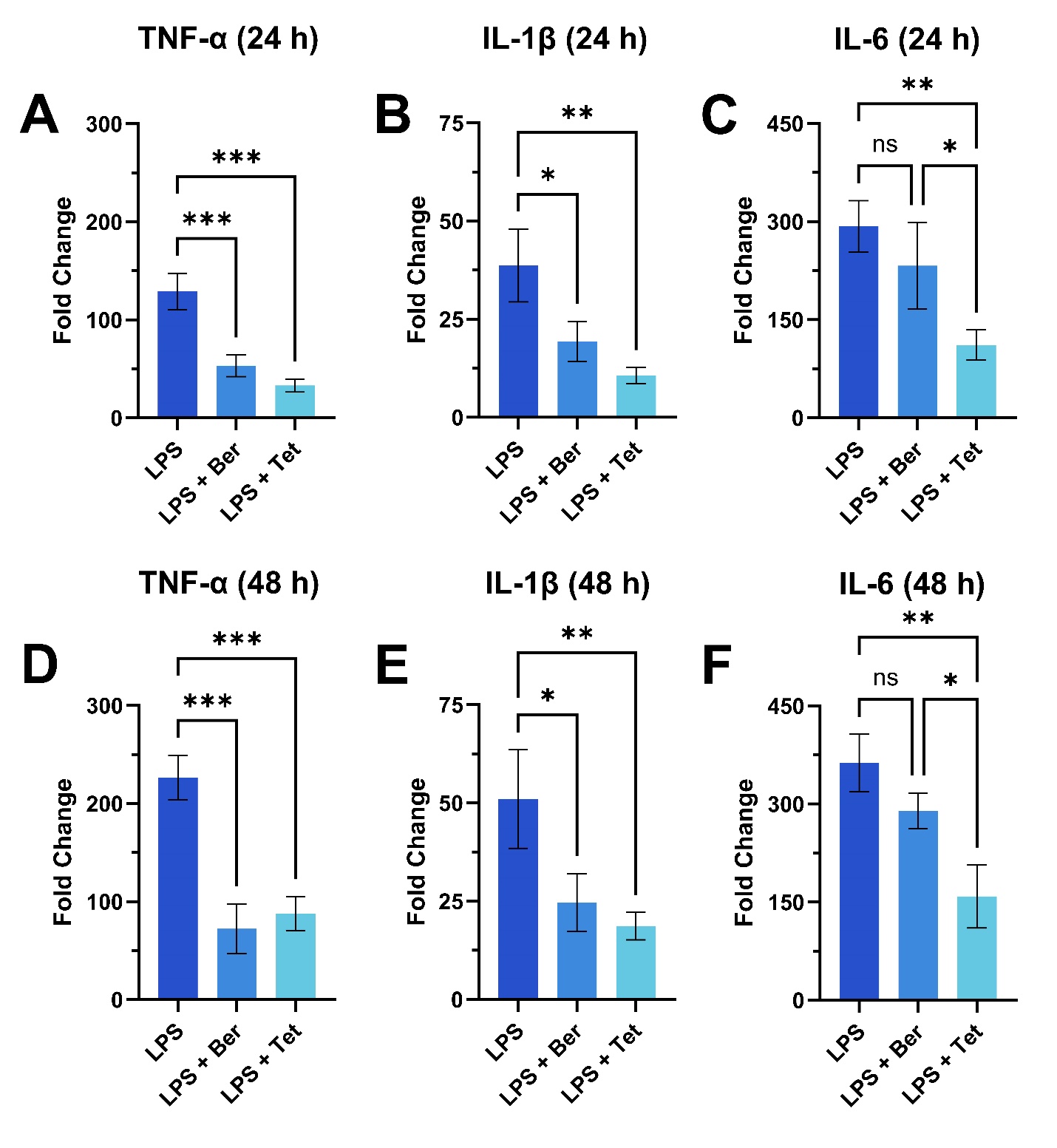


**Fig. S3.** Inhibition of pro-inflammatory cytokine production in LPS-challenged PAMs. Effect of berbamine (Ber) or tetrandrine (Tet) on inhibition of **(A)** TNF-α, **(B)** IL-1β, and **(C)** IL-6 production in LPS-challenged cells after 24 h addition. **(D-F)** Corresponding measurements after 48 h addition. Cytokine measurements were performed by ELISA on collected supernatants. The concentration of berbamine and tetrandrine for the experiments were 50 μM and 25 μM, respectively. LPS means LPS challenge without compound addition while Ber and Tet denote berbamine and tetrandrine, respectively. Results represent the mean ± s.d. of three independent experiments (*n*=3). Significant differences compared to other test groups are denoted by ^*^*p* < 0.05, ^**^*p* < 0.01, and ^***^*p* < 0.001.

**Table S1.** Natural, anti-inflammatory compounds that were tested against ASFV.

| **Name** | **Formula** | **MW (Da)** | **Type** | **Test conc. (µM)** |
| --- | --- | --- | --- | --- |
| (S)-10-Hydroxycamptothecin | C20H16N2O5 | 364.35 | Alkaloids | 50 |
| 10-Gingerol | C21H34O4 | 350.49 | Phenols | 50 |
| 14-Deoxy-11,12-didehydroandrographolide | C20H28O4 | 332.4 | Diterpenoids | 50 |
| 20(R)-Ginsenoside Rh2 | C36H62O8 | 622.88 | Triterpenoids | 50 |
| 3,4',5-Trimethoxystilbene | C17H18O3 | 270.32 | Phenols | 50 |
| 4-Hydroxyisoleucine | C6H13NO3 | 147.17 | Alkaloids | 50 |
| 4-Methylesculetin | C10H8O4 | 192.17 | Coumarins | 50 |
| 5-Hydroxy-3',4',7-trimethoxyflavone | C18H16O6 | 328.3 | Flavonoids | 50 |
| 5-Hydroxytryptophan | C11H12N2O3 | 220.23 | Alkaloids | 50 |
| 6,7-Dihydroxycoumarin | C9H6O4 | 178.14 | Coumarins | 50 |
| 6-Gingerol | C17H26O4 | 294.4 | Phenols | 50 |
| 6-Shogaol | C17H24O3 | 276.37 | Phenols | 50 |
| 8-Gingerol | C19H30O4 | 322.44 | Phenols | 50 |
| 8-O-Acetylshanzhiside methyl ester | C19H28O12 | 448.4 | Iridoids | 50 |
| Abietic acid | C20H30O2 | 302.45 | Diterpenoids | 50 |
| Acacetin | C16H12O5 | 284.3 | Flavonoids | 50 |
| Acetylcorynoline | C23H23NO6 | 409.43 | Alkaloids | 50 |
| Acteoside | C29H36O15 | 624.6 | Phenylpropanoids | 50 |
| Agnuside | C22H26O11 | 466.4 | Iridoids | 50 |
| Alantolactone | C15H20O2 | 232.32 | Sesquiterpenoids | 50 |
| Albiflorin | C23H28O11 | 480.46 | Monoterpenoids | 50 |
| Allicin | C6H10S2O | 162.27 | Miscellaneous | 50 |
| Alliin | C6H11NO3S | 177.22 | Alkaloids | 50 |
| Alnustone | C19H18O | 262.35 | Phenols | 50 |
| Aloin A | C21H22O9 | 418.39 | Anthraquinones | 50 |
| Aloin B | C21H22O9 | 418.39 | Anthraquinones | 50 |
| Aloperine | C15H24N2 | 232.37 | Alkaloids | 50 |
| Alpha-caryophyllene | C15H24 | 204.4 | Sesquiterpenoids | 50 |
| alpha-Cyperone | C15H22O | 218.33 | Sesquiterpenoids | 50 |
| alpha-Mangostin | C24H26O6 | 410.5 | Xanthones | 50 |
| Alpha-Terpineol | C10H18O | 154.25 | Monoterpenoids | 50 |
| Alpinetin | C16H14O4 | 270.3 | Flavonoids | 50 |
| Amphotericin B | C47H73NO17 | 924.07 | Miscellaneous | 50 |
| Amygdalin | C20H27NO11 | 457.4 | Phenols | 50 |
| Andrographolide | C20H30O5 | 350.5 | Diterpenoids | 50 |
| Angelicin | C11H6O3 | 186.2 | Coumarins | 50 |
| Angoroside C | C36H48O19 | 784.75 | Phenylpropanoids | 50 |
| Anhydroicaritin | C21H20O6 | 368.38 | Flavonoids | 50 |
| Anisodamine | C17H23NO4 | 305.37 | Alkaloids | 50 |
| Arbutin | C12H16O7 | 272.26 | Phenols | 50 |
| Arctigenin | C21H24O6 | 372.41 | Lignans | 50 |
| Arctiin | C27H34O11 | 534.55 | Lignans | 50 |
| Artemisinin | C15H22O5 | 282.3 | Sesquiterpenoids | 50 |
| Artesunate | C19H28O8 | 384.42 | Sesquiterpenoids | 50 |
| Asiatic acid | C30H48O5 | 488.7 | Triterpenoids | 50 |
| Asiaticoside | C48H78O19 | 959.12 | Triterpenoids | 50 |
| Asperuloside | C18H22O11 | 414.4 | Iridoids | 50 |
| Astaxanthin | C40H52O4 | 596.85 | Miscellaneous | 50 |
| Astragaloside I | C45H72O16 | 869.1 | Triterpenoids | 50 |
| Astragaloside IV | C41H68O14 | 784.98 | Triterpenoids | 50 |
| Atractylenolide I | C15H18O2 | 230.3 | Sesquiterpenoids | 50 |
| Atractylenolide III | C15H20O3 | 248.32 | Sesquiterpenoids | 50 |
| Aucubin | C15H22O9 | 346.33 | Iridoids | 50 |
| Aurantio-obtusin | C17H14O7 | 330.29 | Anthraquinones | 50 |
| Auraptene | C19H22O3 | 298.4 | Coumarins | 50 |
| Berbamine | C37H40N2O6 | 608.7 | Alkaloids | 50 |
| Berberine | C20H18NO4 | 336.4 | Alkaloids | 50 |
| Berberine hydrochloride | C20H18NO4Cl | 371.81 | Alkaloids | 50 |
| Bergapten | C12H8O4 | 216.2 | Coumarins | 50 |
| Bergenin | C14H16O9 | 328.3 | Phenols | 50 |
| Betaine | C5H11NO2 | 117.15 | Alkaloids | 50 |
| Betulin | C30H50O2 | 442.7 | Triterpenoids | 50 |
| Betulinic acid | C30H48O3 | 456.7 | Triterpenoids | 50 |
| Bisdemethoxycurcumin | C19H16O4 | 308.33 | Phenols | 50 |
| Borneol | C10H18O | 154.25 | Monoterpenoids | 50 |
| Butein | C15H12O5 | 272.3 | Chalcones | 50 |
| Byakangelicol | C17H16O6 | 316.31 | Coumarins | 50 |
| Cannabidiol | C21H30O2 | 314.5 | Phenols | 50 |
| Cardamonin | C16H14O4 | 270.3 | Chalcones | 50 |
| Carnosol | C20H26O4 | 330.42 | Diterpenoids | 50 |
| Catalpol | C15H22O10 | 362.3 | Iridoids | 50 |
| Celastrol | C29H38O4 | 450.61 | Triterpenoids | 50 |
| Cepharanthine | C37H38N2O6 | 606.71 | Alkaloids | 50 |
| Chicoric acid | C22H18O12 | 474.37 | Phenylpropanoids | 50 |
| Chrysophanol | C15H10O4 | 254.2 | Anthraquinones | 50 |
| Cineole | C10H18O | 154.25 | Monoterpenoids | 50 |
| Cinnamic aldehyde | C9H8O | 132.16 | Phenylpropanoids | 50 |
| Coixol | C8H7NO3 | 165.1 | Alkaloids | 50 |
| Columbianadin | C19H20O5 | 328.36 | Coumarins | 50 |
| Coptisine | C19H14NO4 | 320.32 | Alkaloids | 50 |
| Cordycepin | C10H13N5O3 | 251.24 | Alkaloids | 50 |
| Corilagin | C27H22O18 | 634.45 | Phenols | 50 |
| Crebanine | C20H21NO4 | 339.4 | Alkaloids | 50 |
| Crocin | C44H64O24 | 976.96 | Diterpenoids | 50 |
| Crotonoside | C10H13N5O5 | 283.24 | Alkaloids | 50 |
| Cryptotanshinone | C19H20O3 | 296.4 | Diterpenoids | 50 |
| Curcumin | C21H20O6 | 368.4 | Phenols | 50 |
| Curcumol | C15H24O2 | 236.35 | Sesquiterpenoids | 50 |
| D-(-)-Salicin | C13H18O7 | 286.27 | Phenols | 50 |
| Danshensu | C9H10O5 | 198.17 | Phenylpropanoids | 50 |
| Daphnetin | C9H6O4 | 178.14 | Coumarins | 50 |
| Decursin | C19H20O5 | 328.36 | Coumarins | 50 |
| Dehydroabietic acid | C20H28O2 | 300.4 | Diterpenoids | 50 |
| Dehydroandrographolide | C20H28O4 | 332.43 | Diterpenoids | 50 |
| Dehydroandrographolide succinate | C28H36O10 | 532.59 | Diterpenoids | 50 |
| Dehydrocorydalin | C22H24NO4 | 366.44 | Alkaloids | 50 |
| Demethoxycurcumin | C20H18O5 | 338.35 | Phenols | 50 |
| Demethylsuberosin | C14H14O3 | 230.3 | Coumarins | 50 |
| Diammonium glycyrrhizinate | C42H68N2O16 | 857 | Triterpenoids | 50 |
| Dihydrotanshinone I | C18H14O3 | 278.3 | Diterpenoids | 50 |
| Echinocystic acid | C30H48O4 | 472.7 | Triterpenoids | 50 |
| Eleutheroside E | C34H46O18 | 742.73 | Lignans | 50 |
| Ellagic acid | C14H6O8 | 302.2 | Phenols | 50 |
| Embelin | C17H26O4 | 294.38 | Quinones | 50 |
| Emodin | C15H10O5 | 270.2 | Anthraquinones | 50 |
| Emodin-8-beta-D-glucoside | C21H20O10 | 432.41 | Anthraquinones | 50 |
| Eriodictyol | C15H12O6 | 288.25 | Flavonoids | 50 |
| Esculentoside A | C42H66O16 | 826.96 | Triterpenoids | 50 |
| Esculentoside H | C48H76O21 | 989.1 | Triterpenoids | 50 |
| Esculin | C15H16O9 | 340.28 | Coumarins | 50 |
| Eudesmin | C22H26O6 | 386.4 | Lignans | 50 |
| Eupatilin | C18H16O7 | 344.31 | Flavonoids | 50 |
| Euphol | C30H50O | 426.71 | Triterpenoids | 50 |
| Fangchinoline | C37H40N2O6 | 608.71 | Alkaloids | 50 |
| Fargesin | C21H22O6 | 370.39 | Lignans | 50 |
| Flavokawain A | C18H18O5 | 314.3 | Chalcones | 50 |
| Flavokawain B | C17H16O4 | 284.3 | Chalcones | 50 |
| Forskolin | C22H34O7 | 410.5 | Diterpenoids | 50 |
| Forsythoside B | C34H44O19 | 756.7 | Phenylpropanoids | 50 |
| Fumaric acid | C4H4O4 | 116.1 | Miscellaneous | 50 |
| gamma-Mangostin | C23H24O6 | 396.4 | Xanthones | 50 |
| Gastrodin | C13H18O7 | 286.28 | Phenols | 50 |
| Genipin | C11H14O5 | 226.23 | Iridoids | 50 |
| Geniposide | C17H24O10 | 388.4 | Iridoids | 50 |
| Gentiopicroside | C16H20O9 | 356.3 | Iridoids | 50 |
| Geraniin | C41H28O27 | 952.64 | Phenols | 50 |
| Germacrone | C15H22O | 218.34 | Sesquiterpenoids | 50 |
| Ginkgolide B | C20H24O10 | 424.4 | Diterpenoids | 50 |
| Ginsenoside Re | C48H82O18 | 947.15 | Triterpenoids | 50 |
| Ginsenoside Rh1 | C36H62O9 | 638.88 | Triterpenoids | 50 |
| Glaucine | C21H25NO4 | 355.43 | Alkaloids | 25 |
| Glibenclamide | C23H28ClN3O5S | 494 | Alkaloids | 25 |
| Glycoursodeoxycholic acid | C26H43NO5 | 449.63 | Steroids | 50 |
| Glycyrrhizic acid | C42H62O16 | 822.92 | Triterpenoids | 50 |
| Gomisin A | C23H28O7 | 416.5 | Lignans | 50 |
| Gossypin | C21H20O13 | 480.38 | Flavonoids | 50 |
| Harpagide | C15H24O10 | 364.35 | Iridoids | 50 |
| Harpagoside | C24H30O11 | 494.49 | Iridoids | 50 |
| Hesperidin methylchalcone | C29H36O15 | 624.59 | Flavonoids | 50 |
| Hispidulin | C16H12O6 | 300.3 | Flavonoids | 50 |
| Homovanillyl alcohol | C9H12O3 | 168.19 | Phenols | 50 |
| Honokiol | C18H18O2 | 266.34 | Lignans | 50 |
| Hydroxygenkwanin | C16H12O6 | 300.3 | Flavonoids | 50 |
| Hydroxysafflor yellow A | C27H32O16 | 612.53 | Chalcones | 50 |
| Imperatorin | C16H14O4 | 270.3 | Coumarins | 50 |
| Indirubin | C16H10N2O2 | 262.26 | Miscellaneous | 50 |
| Irigenin | C18H16O8 | 360.31 | Flavonoids | 50 |
| Isoacteoside | C29H36O15 | 624.6 | Phenylpropanoids | 50 |
| Isobavachalcone | C20H20O4 | 324.37 | Chalcones | 50 |
| Isofraxidin | C11H10O5 | 222.19 | Coumarins | 50 |
| Isoimperatorin | C16H14O4 | 270.28 | Coumarins | 50 |
| Isoliensinine | C37H42N2O6 | 610.75 | Alkaloids | 50 |
| Isoliquiritigenin | C15H12O4 | 256.3 | Chalcones | 50 |
| Isovitexin | C21H20O10 | 432.4 | Flavonoids | 50 |
| Isoxanthohumol | C21H22O5 | 354.4 | Flavonoids | 50 |
| Juglone | C10H6O3 | 174.16 | Quinones | 50 |
| Karanjin | C18H12O4 | 292.29 | Flavonoids | 50 |
| Kirenol | C20H34O4 | 338.5 | Diterpenoids | 50 |
| Kurarinone | C26H30O6 | 438.5 | Flavonoids | 50 |
| Kynurenic acid | C10H7NO3 | 189.17 | Alkaloids | 50 |
| Licochalcone A | C21H22O4 | 338.4 | Chalcones | 50 |
| Licochalcone B | C16H14O5 | 286.28 | Chalcones | 50 |
| Ligustilide | C12H14O2 | 190.24 | Miscellaneous | 50 |
| Limonin | C26H30O8 | 470.5 | Triterpenoids | 50 |
| Linarin | C28H32O14 | 592.6 | Flavonoids | 50 |
| Loganic acid | C16H24O10 | 376.4 | Iridoids | 50 |
| Loganin | C17H26O10 | 390.4 | Iridoids | 50 |
| Lupeol | C30H50O | 426.7 | Triterpenoids | 50 |
| Lycorine | C16H17NO4 | 287.31 | Alkaloids | 50 |
| Madecassic acid | C30H48O6 | 504.7 | Triterpenoids | 50 |
| Madecassoside | C48H78O20 | 975.13 | Triterpenoids | 50 |
| Magnolin | C23H28O7 | 416.5 | Lignans | 50 |
| Magnolol | C18H18O2 | 266.3 | Lignans | 50 |
| Maltotetraose | C24H42O21 | 666.6 | Miscellaneous | 50 |
| Mangiferin | C19H18O11 | 422.3 | Xanthones | 50 |
| Matrine | C15H24N2O | 248.4 | Alkaloids | 50 |
| Menthone | C10H18O | 154.3 | Monoterpenoids | 50 |
| Methyl ferulate | C11H12O4 | 208.2 | Phenylpropanoids | 50 |
| Methyl salicylate | C8H8O3 | 152.15 | Phenols | 50 |
| Micheliolide | C15H20O3 | 248.3 | Sesquiterpenoids | 50 |
| Mitraphylline | C21H24N2O4 | 368.43 | Alkaloids | 50 |
| Mollugin | C17H16O4 | 284.3 | Quinones | 50 |
| Mollugin | C17H16O4 | 284.3 | Quinones | 50 |
| Monotropein | C16H22O11 | 390.34 | Iridoids | 50 |
| Mulberroside A | C26H32O14 | 568.52 | Phenols | 50 |
| Myrislignan | C21H26O6 | 374.43 | Phenylpropanoids | 50 |
| Naringenin chalcone | C15H12O5 | 272.25 | Chalcones | 50 |
| Neferine | C38H44N2O6 | 624.77 | Alkaloids | 50 |
| Negletein | C16H12O5 | 284.26 | Flavonoids | 50 |
| Neoandrographolide | C26H40O8 | 480.6 | Diterpenoids | 50 |
| Neochlorogenic acid | C16H18O9 | 354.3 | Phenylpropanoids | 50 |
| Neohesperidin | C28H34O15 | 610.56 | Flavonoids | 50 |
| Neomangiferin | C25H28O16 | 584.48 | Xanthones | 50 |
| Nitidine chloride | C21H18ClNO4 | 383.83 | Alkaloids | 50 |
| Nodakenin | C20H24O9 | 408.4 | Coumarins | 50 |
| Nomilin | C28H34O9 | 514.56 | Triterpenoids | 50 |
| Nonivamide | C17H27NO3 | 293.4 | Alkaloids | 50 |
| Nootkatone | C15H22O | 218.3 | Sesquiterpenoids | 50 |
| Norisoboldine | C18H19NO4 | 313.35 | Alkaloids | 50 |
| Notopterol | C21H22O5 | 354.4 | Coumarins | 50 |
| Nuciferine | C19H21NO2 | 295.38 | Alkaloids | 50 |
| Obtusifolin | C16H12O5 | 284.27 | Anthraquinones | 50 |
| Oleanolic acid | C30H48O3 | 456.7 | Triterpenoids | 50 |
| Oxymatrine | C15H24N2O2 | 264.4 | Alkaloids | 50 |
| Oxypeucedanin | C16H14O5 | 286.28 | Coumarins | 50 |
| Oxysophocarpine | C15H22N2O2 | 262.4 | Alkaloids | 50 |
| Oxysophoridine | C15H24N2O2 | 264.36 | Alkaloids | 50 |
| Pachymic acid | C33H52O5 | 528.76 | Triterpenoids | 50 |
| Paeoniflorin | C23H28O11 | 480.45 | Monoterpenoids | 50 |
| Paeonol | C9H10O3 | 166.2 | Phenols | 50 |
| Parthenolide | C15H20O3 | 248.3 | Sesquiterpenoids | 50 |
| Pectolinarin | C29H34O15 | 622.57 | Flavonoids | 50 |
| Peimine | C27H45NO3 | 431.66 | Alkaloids | 50 |
| Peiminine | C27H43NO3 | 429.64 | Alkaloids | 50 |
| Phellopterin | C17H16O5 | 300.3 | Coumarins | 50 |
| Phillyrin | C27H34O11 | 534.56 | Lignans | 50 |
| Phlorizin | C21H24O10 | 436.4 | Chalcones | 50 |
| Phytol | C20H40O | 296.5 | Diterpenoids | 50 |
| Piceatannol | C14H12O4 | 244.2 | Phenols | 50 |
| Picroside II | C23H28O13 | 512.47 | Iridoids | 50 |
| Pimpinellin | C13H10O5 | 246.2 | Coumarins | 50 |
| Pinoresinol | C20H22O6 | 358.4 | Lignans | 50 |
| Piperine | C17H19NO3 | 285.34 | Alkaloids | 50 |
| Platycodin D | C57H92O28 | 1225.33 | Triterpenoids | 50 |
| Plumbagin | C11H8O3 | 188.17 | Quinones | 50 |
| Pogostone | C12H16O4 | 224.26 | Miscellaneous | 50 |
| Polydatin | C20H22O8 | 390.4 | Polyphenols | 50 |
| Poncirin | C28H34O14 | 594.56 | Flavonoids | 50 |
| Praeruptorin A | C21H22O7 | 386.4 | Coumarins | 50 |
| Prim-O-glucosylcimifugin | C22H28O11 | 468.45 | Polyphenols | 50 |
| Pristimerin | C30H40O4 | 464.64 | Triterpenoids | 50 |
| Proanthocyanidins | C30H26O13 | 594.52 | Flavonoids | 50 |
| Pseudoginsenoside F11 | C42H72O14 | 801.01 | Triterpenoids | 50 |
| Psoralidin | C20H16O5 | 336.34 | Coumarins | 50 |
| Pterostilbene | C16H16O3 | 256.3 | Phenols | 50 |
| Punicalagin | C48H28O30 | 1084.72 | Phenols | 25 |
| Rhein | C15H8O6 | 284.21 | Anthraquinones | 50 |
| Rhoifolin | C27H30O14 | 578.5 | Flavonoids | 50 |
| Rhynchophylline | C22H28N2O4 | 384.47 | Alkaloids | 50 |
| Riboflavine | C17H20N4O6 | 376.36 | Alkaloids | 50 |
| Rosmarinic acid | C18H16O8 | 360.31 | Phenylpropanoids | 50 |
| Rutaecarpine | C18H13N3O | 287.3 | Alkaloids | 50 |
| Saikosaponin A | C42H68O13 | 780.99 | Triterpenoids | 50 |
| Saikosaponin D | C42H68O13 | 780.99 | Triterpenoids | 50 |
| Salidroside | C14H20O7 | 300.3 | Phenols | 50 |
| Salvianolic acid A | C26H22O10 | 494.45 | Phenylpropanoids | 50 |
| Salvianolic acid B | C36H30O16 | 718.62 | Phenylpropanoids | 50 |
| Sanguinarine | C20H14NO4 | 332.3 | Alkaloids | 50 |
| Sauchinone | C20H20O6 | 356.36 | Diterpenoids | 50 |
| Schisandrol A | C24H32O7 | 432.5 | Lignans | 50 |
| Schisantherin A | C30H32O9 | 536.56 | Lignans | 50 |
| Schizandrin A | C24H32O6 | 416.51 | Lignans | 50 |
| Scoparone | C11H10O4 | 206.2 | Coumarins | 50 |
| Scopoletin | C10H8O4 | 192.2 | Coumarins | 50 |
| Secoisolariciresinol Diglucoside | C32H46O16 | 686.71 | Lignans | 50 |
| Senegenin | C30H45ClO6 | 537.14 | Triterpenoids | 50 |
| Sesamin | C20H18O6 | 354.4 | Lignans | 50 |
| Shikonine | C16H16O5 | 288.3 | Quinones | 50 |
| Sinapic acid | C11H12O5 | 224.2 | Phenylpropanoids | 50 |
| Sinomenine | C19H23NO4 | 329.38 | Alkaloids | 50 |
| Sophocarpine | C15H22N2O | 246.4 | Alkaloids | 50 |
| Sophoridine | C15H24N2O | 248.4 | Alkaloids | 50 |
| Stachyose | C24H42O21 | 666.57 | Miscellaneous | 50 |
| Stevioside | C38H60O18 | 804.88 | Diterpenoids | 50 |
| Suberosin | C15H16O3 | 244.3 | Coumarins | 50 |
| Swertiamarin | C16H22O10 | 374.3 | Iridoids | 50 |
| Syringic acid | C9H10O5 | 198.2 | Phenols | 50 |
| Syringin | C17H24O9 | 372.4 | Phenylpropanoids | 50 |
| Tanshinone I | C18H12O3 | 276.3 | Diterpenoids | 50 |
| Tanshinone IIA | C19H18O3 | 294.4 | Diterpenoids | 50 |
| Tenuifoliside A | C31H38O17 | 682.6 | Phenylpropanoids | 50 |
| Tetrahydrocurcumin | C21H24O6 | 372.41 | Phenols | 50 |
| Tetramethylpyrazine | C8H12N2 | 136.2 | Alkaloids | 50 |
| Tetrandrine | C38H42N2O6 | 622.76 | Alkaloids | 25 |
| Theophylline | C7H8N4O2 | 180.16 | Alkaloids | 50 |
| Tiliroside | C30H26O13 | 594.5 | Flavonoids | 50 |
| Timosaponin A3 | C39H64O13 | 740.92 | Steroids | 50 |
| Tomatidine | C27H45NO2 | 415.65 | Alkaloids | 50 |
| trans-Caryophyllene | C15H24 | 204.36 | Sesquiterpenoids | 50 |
| Trifolirhizin | C22H22O10 | 446.4 | Flavonoids | 50 |
| Trilobatin | C21H24O10 | 436.4 | Chalcones | 50 |
| Trimethylapigenin | C18H16O5 | 312.32 | Flavonoids | 50 |
| Trimethylgallic acid | C10H12O5 | 212.2 | Phenols | 50 |
| Triptolide | C20H24O6 | 360.4 | Diterpenoids | 50 |
| Tussilagone | C23H34O5 | 390.51 | Sesquiterpenoids | 50 |
| Ursolic acid | C30H48O3 | 456.7 | Triterpenoids | 50 |
| Usnic acid | C18H16O7 | 344.3 | Phenols | 50 |
| Vanillic acid | C8H8O4 | 168.2 | Phenols | 50 |
| Vinpocetine | C22H26N2O2 | 350.45 | Alkaloids | 50 |
| Vitexicarpin | C19H18O8 | 374.34 | Flavonoids | 50 |
| Wedelolactone | C16H10O7 | 314.3 | Coumarins | 50 |
| Wogonoside | C22H20O11 | 460.39 | Flavonoids | 25 |
| Xanthohumol | C21H22O5 | 354.4 | Chalcones | 50 |
| Xanthotoxol | C11H6O4 | 202.2 | Coumarins | 50 |
| Zerumbone | C15H22O | 218.3 | Sesquiterpenoids | 50 |
| Zingerone | C11H14O3 | 194.23 | Phenols | 50 |
